# A Meta-Analysis on Presynaptic Changes in Alzheimer’s Disease

**DOI:** 10.3233/JAD-231034

**Published:** 2024-01-02

**Authors:** Anne Anschuetz, Karima Schwab, Charles R. Harrington, Claude M. Wischik, Gernot Riedel

**Affiliations:** a School of Medicine, Medical Sciences and Nutrition, University of Aberdeen, Aberdeen, UK; b TauRx Therapeutics Ltd., Aberdeen, UK

**Keywords:** Alzheimer’s disease, meta-analysis, presynaptic terminals, synaptic vesicles

## Abstract

**Background:**

A key aspect of synaptic dysfunction in Alzheimer’s disease (AD) is loss of synaptic proteins. Previous publications showed that the presynaptic machinery is more strongly affected than postsynaptic proteins. However, it has also been reported that presynaptic protein loss is highly variable and shows region- and protein-specificity.

**Objective:**

The objective of this meta-analysis was to provide an update on the available literature and to further characterize patterns of presynaptic protein loss in AD.

**Methods:**

Systematic literature search was conducted for studies published between 2015–2022 which quantified presynaptic proteins in postmortem tissue from AD patients and healthy controls. Three-level random effects meta-analyses of twenty-two identified studies was performed to characterize overall presynaptic protein loss and changes in specific regions, proteins, protein families, and functional categories.

**Results:**

Meta-analysis confirmed overall loss of presynaptic proteins in AD patients. Subgroup analysis revealed region specificity of protein loss, with largest effects in temporal and frontal cortex. Results concerning different groups of proteins were also highly variable. Strongest and most consistently affected was the family of synaptosome associated proteins, especially SNAP25. Among the most severely affected were proteins regulating dense core vesicle exocytosis and the synaptic vesicle cycle.

**Conclusions:**

Results confirm previous literature related to presynaptic protein loss in AD patients and provide further in-depth characterization of most affected proteins and presynaptic functions.

## INTRODUCTION

Multiple lines of evidence suggest that synaptic dysfunction is a key early pathological feature of Alzheimer’s disease (AD) and that it correlates with the emergence and progression of cognitive impairment [1–5]. Synaptic dysfunction includes dendrite abnormalities, enlarged presynaptic terminals and synaptic vesicles, as well as overall synapse loss [3, 6–9]. Synapse loss occurs in patients with mild cognitive impairment and progresses with disease severity [3, 5] concomitant with alterations in level and function of synaptic proteins [4]. Both events are presumably triggered by the pre- and postsynaptic accumulation of pathological forms of tau and amyloid-β [9–13], eventually leading to dysfunctional synapses [14–16].

A substantial heterogeneity in terms of regional differences of synaptic protein alterations/overall synapse loss was suggested early on [17]. In a recent review of 3D electron microscopy studies, associations between synapse and neuron loss differed between brain regions suggesting varying synapse vulnerability [18]. Indeed, the hippocampus seems to be the most affected structure, while neocortical regions show synapse loss only at later disease stages and the entorhinal cortex presents little to no loss of synapses even at advanced stages [3, 19, 20]. A meta-analysis confirmed that presynaptic proteins were consistently more affected than postsynaptic proteins [21]. In this publication, we have sought to further characterize these changes in AD and provide an update of the literature published since the previous meta-analysis with a focus on presynaptic proteins as the most strongly affected early synaptic markers. Thus, a systematic literature search from 2015–2022 for publications measuring proteins with function at the presynapse, as defined by SynGo (https://www.syngoportal.org/) [22], in AD and control tissue was conducted and a meta-analysis performed on data from 22 studies. Due to the paucity of data, individual subcortical regions could not be distinguished, and so region-specific results are limited to cortical structures. The analysis here provides further support that presynaptic protein changes in AD are highly heterogeneous. While there was an overall reduction in presynaptic proteins in AD patients, areas such as temporal and frontal cortex were more severely affected than others. Synaptic proteins and functional categories also showed heterogeneity.

## MATERIALS AND METHODS

### Search strategy

Medline, Embase, and PubMed databases were searched for articles reporting brain presynaptic protein levels in AD patients and animal models compared to healthy controls. Here, only the results on human patients are reported. Databases were searched for the following keywords in abstract and title:

presynaptic marker *or* presynaptic protein *or* synaptic marker *or* synaptic protein *or* proteome

*and*

AD *or* Alzheimer

**Fig. 1:**
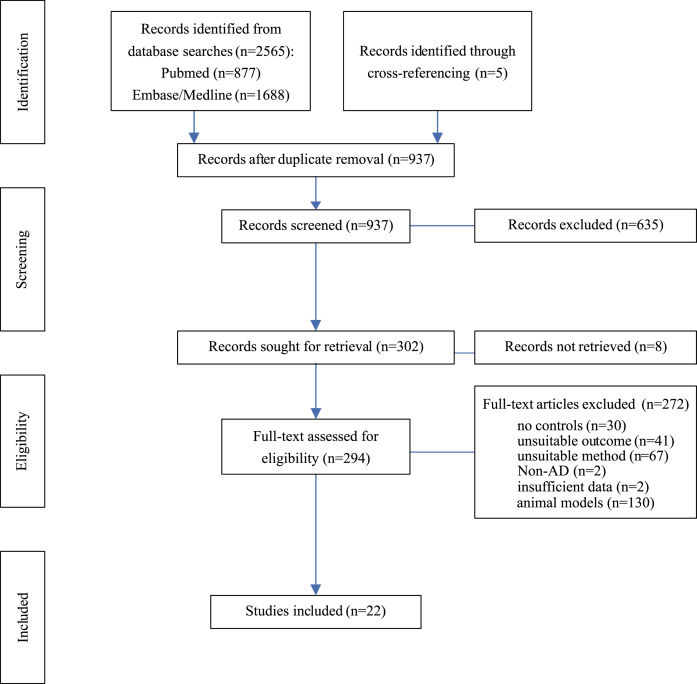
Flow Chart. Diagram of systematic literature search and progress of selection of articles. AD, Alzheimer’s disease; Left column indicates levels of activity.

The search was restricted to publications since 2015 and filters were used to remove non-English publications, reviews, and conference abstracts. Database search in February 2022 resulted in 2,565 matches (Fig. 1). The systematic review tool Rayyan (https://www.rayyan.ai) [23] was used for screening. Duplicates from Medline and Embase were removed automatically (*n* = 769) and further duplicates with PubMed recognized by Rayyan were omitted manually (*n* = 864). Title and abstracts were screened for eligibility in Rayyan according to predefined inclusion and exclusion criteria. Inclusion criteria were comparison of an AD population to healthy controls, and quantification of proteins in brain with functions at the presynapse, as defined by SynGo (https://www.syngoportal.org/) [22]. Reviews, conference abstracts and publications not including an AD population, lacking controls, or only measuring gene or mRNA expression were excluded. Full texts for eligible studies were retrieved in pdf format; full texts for eight studies were inaccessible and not provided by authors upon request. Relevant studies were searched for cross-references, resulting in identification of a further five studies that were then included. Database search was repeated in August 2022, with no additional studies on patient cohorts being identified.

For patient analyses, only reports quantifying presynaptic protein levels in postmortem brain tissue from AD patients and healthy controls were included. Outcomes not suitable for this analysis such as postsynaptic protein quantification or reports quantifying proteins in tissues other than brain were excluded. Global proteomic approaches on global brain tissue analyses were not considered, while those on synaptosomal or synaptic enriched fractions were included. Studies on diseases other than AD and those lacking healthy controls were not considered. If summary statistics of presynaptic protein expression in one or both groups were not available and could not be obtained from authors, then such studies were removed.

### Data extraction

Twenty-two publications fulfilled all inclusion criteria [24–45] (Fig. 1). Study characteristics were extracted, including proteins measured, method of quantification, brain area, tissue source, sample sizes, age, gender distribution and *post-mortem* interval (Supplementary Table 2). Not all studies reported all relevant study characteristics (Supplementary Table 1). Where individual group demographics could not be extracted, overall sample characteristics were selected.

Numerical data on protein expression were extracted from full texts but, if not available, numerical values were extracted via WebPlotDigitizer (https://automeris.io/WebPlotDigitizer/) [46] from figures (Supplementary Table 1). Missing data were requested from authors and included if provided. Note that Hesse et al. [32] pooled their samples before quantification according to group status (AD or control) and *APOE* genotype (*APOE* *ɛ*3/*ɛ*3 or *APOE* *ɛ*3/*ɛ*4) which resulted in two measurements per group for all proteins and brain structures. For meta-analysis, n for each group, AD and control, was thus two.

### Data analysis

Most studies included here reported multiple effect sizes such as analysis of several proteins or brain areas in multiple groups or application of various methods. This effect size multiplicity can result in challenges for meta-analyses as the same subjects contribute to multiple effect sizes. Several strategies for addressing this were applied depending on the source of multiplicity including selecting one effect size based on decision rules; averaging effect size and conducting multilevel analysis with nested effects sizes (see for overview of strategies [47]).

Several studies analyzed multiple groups or did not separate their sample into dichotomous categories of AD and non-AD/controls. In such cases, decision rules were applied to select the relevant outcomes for analysis. For studies in which additional neurodegenerative diseases other than AD were included, only control and AD groups were extracted for analysis. When subjects were grouped according to Braak stages, the group with the lowest stage (maximum Braak II) was considered as control and the cohort with most severe Braak stage reported (minimum Braak IV) was included as AD group. For studies reporting multiple AD groups such as familial and sporadic AD, the group with demographic characteristics best matching the remaining study cohorts was selected. Buchanan et al. [26] reported results for their full sample as well as when removing five controls with overt non-AD-related pathologies. Here, only the latter results were considered. In cases reporting different methods to quantify the same proteins in the same sample, only one method was included for analysis. For instance, in Bereczki et al. [24] enzyme-linked immunosorbent assay (ELISA) and western blotting were applied for protein quantification. We selected ELISA data as control and AD group mean and standard deviation was available in the text. By contrast, Kurbatskaya et al. [35] reported protein levels quantified by western blotting using two different loading controls, neuron-specific enolase (NSE) and β-actin. Only expression data using the NSE were included for analysis here. While Nyarko and colleagues [37] reported expression of solute carrier family 18 member A2 (SLC18A2) in different glycosylation states we choose total SLC18A2 expression per subject.

For several reports expression data was aggregated by computing averages of the effect sizes with inverse-variance weighting. Our main goal was to estimate the brain-wide effect of AD on presynaptic protein levels as well as investigating overall effects for main brain areas and proteins where applicable. Therefore, it was deemed acceptable to aggregate data to one overall effect size per area or protein where expression was analyzed for subregions and protein isoforms. Haytural and colleagues [31] measured five proteins in ten hippocampal subregions, which would result in 50 individual effect sizes. This overestimation of effect sizes was reduced by combining effect sizes for each protein within the dentate gyrus and *cornu ammonis*. Similarly, Hoshi et al. [33] and Yamazaki et al. [45] analyzed subregions in temporal and frontal cortex, respectively, and individual effect sizes were combined as a single effect size. Ramos-Miguel et al. [39] measured long and short splice variants of syntaxin-binding protein 1 (STXBP1) and this was aggregated to one overall effect size for STXBP1. The same approach was taken for the proteomic analyses of Carlyle et al. [27] and Hesse et al. [32], where multiple isoforms were reported.

Sensitivity analysis was conducted for several approaches to determine whether they would alter the meta-analysis results and it was revealed that they do not. Finally, remaining effect size multiplicity was due to analyses of several proteins and/or several brain regions within one study and this was accounted for by conducting multilevel meta-analyses where multiple effect sizes were nested within one study. This approach has the advantage of allowing estimation of variance of effect sizes within (Level 2) and between studies (Level 3) [48].

### Primary analysis

The primary analysis was performed with *metafor* [49], *meta* [50], and *altmeta* [51] packages in R Studio [52]. The standardized mean difference adjusted for small sample sizes (Hedges’ g) was used as a measure of effect size [53]. When more than one protein or more than one brain structure were investigated, each effect size was added for analysis individually. A multilevel meta-analysis was conducted on all available effect sizes for an overall effect of AD on presynaptic proteins across the whole brain. As substantial between-study heterogeneity had been predicted, a random-effects approach was applied for this meta-analysis [54]. Heterogeneity between studies was calculated using Q-test and I^2^ statistic[55, 56].

### Secondary analysis

For secondary analyses, effects for different brain areas, individual proteins and presynaptic functions were considered. A multilevel random-effects meta-analysis was performed if five or more independent studies were available, as recommended previously [57]. Despite reduced statistical power, some relevant exploratory analyses were also performed on brain regions, proteins, and functional groups with lower study numbers. Multilevel random-effects meta-analyses were performed on cortical regions as subcortical regions were only reported in one study. For analysis of specific presynaptic functions, proteins were annotated with their respective functional term extracted from SynGo [22]. Function annotations included regulation of presynaptic cytosolic calcium levels, regulation of presynaptic membrane potential, presynaptic endocytosis, synaptic vesicle cycle, presynaptic dense core vesicle exocytosis, neurotransmitter uptake, neurotransmitter reuptake, presynaptic chaperone-mediated protein folding and presynaptic signaling pathways. As many proteins have multiple presynaptic functions and would therefore contribute to the analysis several times, separate multilevel random-effects meta-analyses were performed for each functional term if data from five or more independent studies were available.

### Sensitivity analysis

Effect sizes within studies might be correlated especially since they stem from the same subjects. The extent of this correlation was not known; therefore, a sensitivity analysis was performed by running multilevel meta-analyses with values for within-study effect sizes correlating between 0 and 0.99. In the primary analysis, one study had a remarkably high negative effect size; a repeat analysis was performed after outlier removal. Additionally, several reports came from the same research team and to account for this, a multilevel meta-analysis was conducted where individual effect sizes were nested within ‘research team’ instead of within ‘individualstudy’.

### Publication bias

When using the standardized mean difference as the effect size proxy, effect size and its standard error are not independent. This would cause funnel plot distortion when plotting effect size against standard error [58, 59]. Therefore, to assess publication bias, funnel plots were generated by displaying effect size against sample size as a measure of precision as recommended in Zwetsloot et al. [59]. Furthermore, the formula suggested by Pustejovsky and Rogers [58] to conduct Eggers’ test using a modified version of standard error was applied to reveal funnel plot asymmetry.

### Quality assessment

The meta-analysis included 22 studies and examined them according to case and control definition, comparability of groups, methodology and outcome reporting. For case and control definition a maximum of two points could be achieved respectively if based on clinical and neuropathological assessment. One point was given if at least neuropathology was assessed and none when information was lacking. A maximum of two points was awarded if criteria were implemented consistently across all subjects including AD diagnostic criteria or neuropathology scales as well as exclusion criteria such as absence of other diseases. One point was given if at least exclusion criteria were consistently used. For group comparability 0–2 points were awarded depending on how well-matched AD and control groups were as well as one point if groups were overall comparable other than the presence or absence of AD. One point each was awarded for appropriate methods of quantification, appropriate statistical analysis, blinding of samples for protein quantification and reporting of data in sufficient detail to allow extraction of group mean and standard deviation from text or figures. Scores were visualized in a color coded chart and percentage of points achieved was calculated.

## RESULTS

To determine changes in protein levels at the presynapse in AD, databases were searched to identify publications quantifying presynaptic proteins in brain samples from AD patients and healthy controls and a meta-analysis was conducted on studies matching inclusion criteria. Database searches on PubMed, Medline, and Embase returned 2,565 records (Fig. 1). An additional five articles were identified through cross-referencing. After removing duplicates from search results, 937 records remained for title and abstract screening; 635 articles did not meet inclusion criteria and a further eight articles had to be excluded as no full text could be retrieved. The full text of the remaining 294 articles were assessed for eligibility. For human studies, 22 met all inclusion criteria and were selected formeta-analysis.

Overall, presynaptic protein measurements from 17 different brain areas and 223 individual proteins were included (Supplementary Table 2). One publication analyzed subcortical areas and the remaining 21 studies included only cortical structures. Western blotting was most frequently used for protein quantification, followed by immunohistochemistry and ELISA. Three studies used mass spectrometry-based approaches including IP-MS (immunoprecipitation mass spectrometry), LC-MS/MS (liquid chromatography tandem mass spectrometry), and LC-MS^3^ (liquid chromatography tandem mass spectrometry cubed). The latter two were conducted on synaptoneurosomal and synaptic-enriched fractions, respectively. When considering all included publications, over 400 control and AD samples were analyzed. However, some studies acquired their samples from the same brain banks, therefore the subjects contributing their brain tissue may overlap across studies.

### Primary analysis

**Fig. 2:**
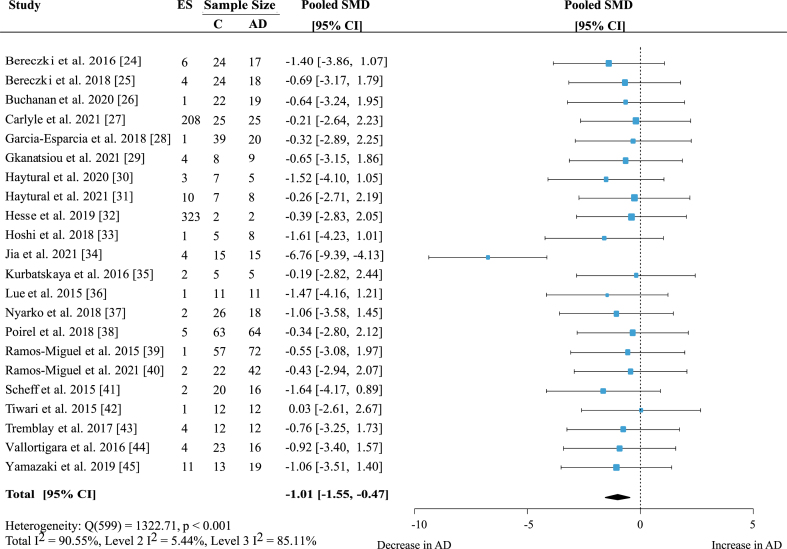
Presynaptic protein changes in AD. Forest plot of three-level random effects meta-analysis of all effect sizes in 22 included studies. Primary analysis revealed a significant loss of presynaptic proteins in AD (*p* < *0.001*). For visualization of the large amount of data included in this analysis, effect sizes were aggregated to one value per study to generate the forest plot. Size of effect size symbol represents its weight. Sample sizes represent the maximum n per group contributing to the analysis for each study. AD, Alzheimer’s disease; CI, confidence interval; C, Control; ES, effect size; SMD, standardized mean difference. Note the abnormally low values of Jia et al. (2021) [34]; only one study [42] did not report a decline in protein levels in AD. Dashed line depicts no difference between control and AD cohort.

For the primary analysis, a random-effects meta-analysis on the 22 human studies was performed including all individual proteins and brain regions. As most studies provided more than one presynaptic protein measurement, three-level analysis was performed with effect size nested within studies. Meta-analysis showed a significant decrease of presynaptic proteins in AD subjects compared to healthy controls (Fig. 2; effect size: –1.01; 95% Confidence Interval (CI): –1.55, –0.47; *p* < 0.001). Heterogeneity was very high, with an I^2^ of 90.55%. However, intra-study heterogeneity was low (Level 2 I^2^: 5.44%) and most of the overall heterogeneity came from between-study variation (Level 3 I^2^: 85.11%). Although the magnitude of effect sizes varied, all studies apart from one reported a decrease in levels of presynaptic protein in AD patients.

### Sensitivity analysis

As it was not known whether effect sizes within studies were correlated and to what extent, a sensitivity analysis with multiple values for effect size correlation was conducted. When using values between 0.1 and 0.99 for intra-study effect size correlation, the overall result of the meta-analysis varied between –1.01 and –1.17 but the effect remained significant for all analyses (*p* < 0.001 for all) (Supplementary Table 3).

A sensitivity analysis was conducted to account for multiple studies from the same research team, whereby instead of clustering effect sizes at the study level, they were clustered within publications by the same research teams. The outcome was similar and showed a significant decrease of presynaptic proteins in AD (effect size: –1.02; 95% CI: –1.64, –0.4; *p* < 0.001) (Supplementary Table 3).

### Outlier removal

When removing the study by Jia et al. [34] with a very large negative effect size (see Fig. 2), the result indicated a smaller overall decrease of presynaptic proteins in AD, but the effect remained significant (effect size: –0.72; 95% CI: –0.93, –0.52; *p* < 0.001) (Supplementary Table 3). The heterogeneity was much lower and was made up of similar amounts of within- and between-study heterogeneity (Total I^2^: 58.73; Level 2 I^2^: 23.97%; Level 3 I^2^: 34.75%). This indicates that in the remaining 21 studies heterogeneity was moderate on all levels and that the outlier study not only affected the overall result but also added substantial inter-study heterogeneity.

### Quality assessment and publication bias

**Fig. 3:**
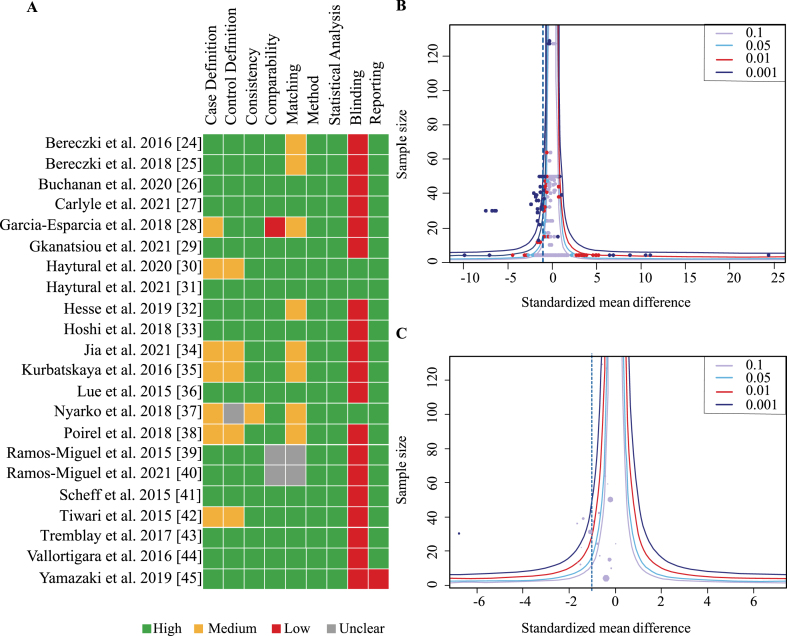
Assessment of study quality and publication bias. A) Studies were assessed for quality according to 9 criteria scored between 0–2 for not present/fully reported. Heat map indicates in green boxes maximal points, orange half maximal points and red indicates no points. B) Funnel plot of included studies with all effect sizes in each study plotted against the sample size (ordinate). Dashed line indicates total effect size of all studies. C) Funnel plot of included studies with aggregated effect size for each study plotted against sample size (ordinate). Number of individual effect sizes is reflected in size of dots. Test for funnel plot asymmetry indicated no publication bias (all effect sizes: *p* = 0.12; Aggregated data: *p* = 0.34).

Overall, nine categories were defined and rated for each publication according to a scale from 0–2 (see Methods). Most studies achieved a moderate to high score in all categories (Fig. 3A). However, only three studies reported that outcome assessors were blinded to group status. Overall scores were moderate to high for all studies with no publication receiving less than 60%. No study was excluded due to low quality.

Funnel plots including all effects sizes from each study as well as one aggregated effect size per study were generated and assessed for publication bias (Fig. 3B, C). Neither showed asymmetry on visual inspection and this was confirmed by the linear regression analysis for funnel plot asymmetry suggesting absence of publication bias (all effect sizes: *p* = 0.12; aggregated data: *p* = 0.34).

### Global changes in presynaptic proteins are region-specific

**Fig. 4:**
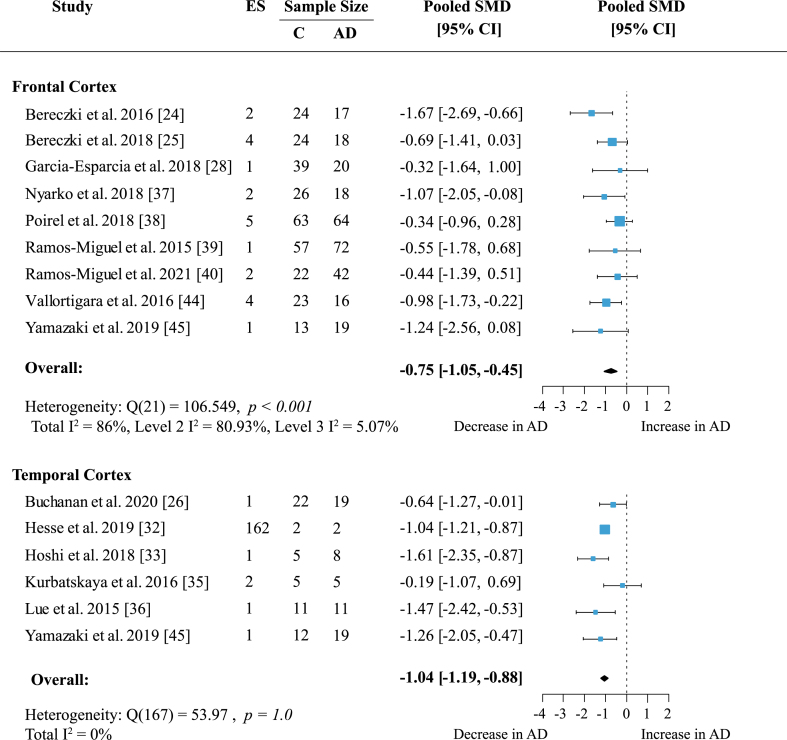
Presynaptic protein changes in cortical regions. Forest plot of three-level random effects meta-analysis on cortical areas. There was significant loss of presynaptic proteins in frontal and temporal cortex (ps < 0.001) in AD. Effect sizes were aggregated to one value per study. Size of effect size symbol represents its weight. Sample sizes represent the maximum n per group contributing to the analysis for each study. Dashed line depicts no difference between control and AD cohort. AD, Alzheimer’s disease, CI, confidence interval; C, Control; ES, effect size; SMD, standardized mean difference.

To determine whether presynaptic protein loss in AD was region-specific, a separate meta-analysis was conducted for available cortical areas. Data from more than five independent datasets was only available for frontal and temporal cortex. Frontal cortex included data on 19 proteins measured across nine studies. In temporal cortex 162 proteins were quantified in six studies. Presynaptic protein loss in AD compared to healthy subjects was higher in the temporal cortex (Fig. 4; effect size: –1.04; 95% CI: –1.19, –0.88; *p* < 0.001) than in frontal cortex (Fig. 4; effect size: –0.75; 95% CI: –1.05, –0.45; *p* < 0.001). Although the magnitude of protein loss varied, especially in the frontal cortex, all reports indicated a genuine loss of presynaptic protein levels in AD. In the temporal cortex, most reports (five out of six) showed a prominent decrease of protein levels in AD of one standardized mean difference or more relative to controls. Heterogeneity in the temporal cortex was not significant. Meanwhile, heterogeneity remained high in the frontal cortex analysis (Total I^2^: 86%) which was largely due to within-study variation (Level 2 I^2^: 80.93%; Level 3 I^2^: 5.07%).

We next performed an exploratory meta-analysis for the remaining cortical structures despite low number of reports. The magnitude of presynaptic protein loss was similar to temporal cortex in parietal cortex (Table 1; effect size: –0.92; 95% CI: –1.65, –0.19; *p* = 0.014) and cingulate gyrus (Table 1; effect size: –1.07; 95% CI: –1.67, –0.47; *p* < 0.001). Data was available in three studies each and included 209 proteins measured for parietal cortex and four proteins measured for cingulate gyrus. Heterogeneity was not significant in cingulate gyrus (*p* = 0.09). In parietal cortex heterogeneity was low within studies (Level 2 I^2^: 15.18%) and moderate between studies (Level 3 I^2^71.66%). Meanwhile, effects in entorhinal cortex, occipital cortex, and hippocampal formation (HPF, including *cornu ammonis* and dentate gyrus) were not significant (Table 1).

### Reduced levels of presynaptic proteins in AD cohorts are protein-specific

**Fig. 5:**
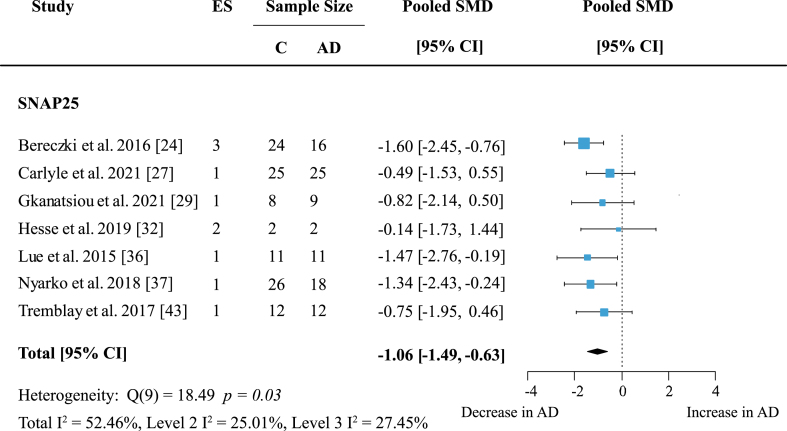
SNAP25 loss in AD. Forest plot of three-level random effects meta-analysis on SNAP25 levels. A reliable decrease in AD (*p* < 0.001) was confirmed. Effect sizes were aggregated to one value per study. Size of effect size symbol represents its weight. Sample sizes represent the maximum n per group contributing to the analysis for each study. Dashed line depicts no difference between control and AD cohort. AD, Alzheimer’s disease, CI, confidence interval; C, Control; ES, effect size; SMD, standardized mean difference; SNAP25, synaptosome associated protein 25.

To reveal whether there are protein-specific effects, proteins, and protein families with effect sizes from at least five independent studies were assessed. Synaptosome associated proteins (SNAPs) were quantified in five brain areas across seven studies. Meta-analysis confirmed a significant decrease in SNAP proteins (Supplementary Figure 1; effect size: –0.90; 95% CI: –1.4, –0.4; *p* < 0.001). The overall effect was strongest for SNAP25 alone (Fig. 5; effect size: –1.06; 95% CI: –1.49, –0.63; *p* < 0.001).

Both analyses showed moderate heterogeneity with I^2^ of 66% and 52.46% respectively. However, for SNAP25 heterogeneity was very similar within- and between studies (Level 2 I^2^: 25.01%; Level 3 I^2^: 27.45%) whereas in the analysis of all SNAP proteins heterogeneity was mainly due to between-study variation (Level 2 I^2^: 13.55%; Level 3 I^2^: 51.32%). All but one report [32] showed a moderate to large decrease of SNAP25 levels in AD patients.

**Fig. 6:**
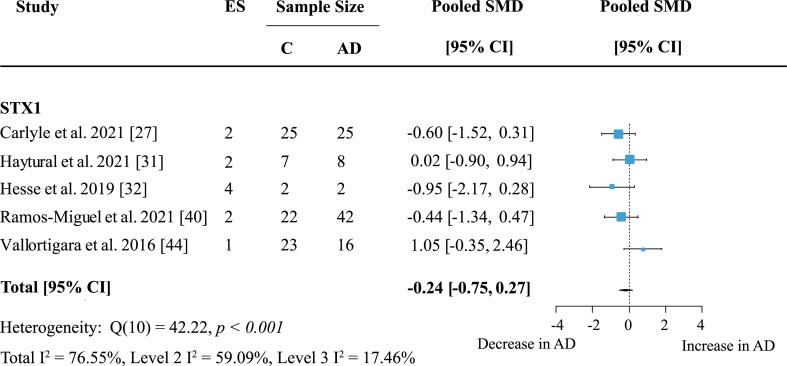
Syntaxin 1 loss in AD. Forest plot of three-level random effects meta-analysis on syntaxin 1 levels. No significant decrease in AD (*p* = 0.35) was obtained. Effect sizes were aggregated to one value per study. Size of effect size symbol represents its weight. Sample sizes represent the maximum n per group contributing to the analysis for each study. Dashed line depicts no difference between control and AD cohort. AD, Alzheimer’s disease, CI, confidence interval; C, Control; ES, effect size; SMD, standardized mean difference; STX1, Syntaxin.

The syntaxin (STX) family showed no significant loss in AD (Supplementary Figure 2). Included in this analysis were six studies measuring syntaxin proteins in five areas. Syntaxin 1 (STX1) was the most frequently analyzed protein in this family and was mentioned in five publications across five brain regions. Syntaxin 1, including isoforms STX1A and STX1B also showed no significant loss in AD subjects compared to controls (Fig. 6). Synaptotagmins (SYTs) were measured in five publications, but no overall change was found (Table 1).

**Fig. 7:**
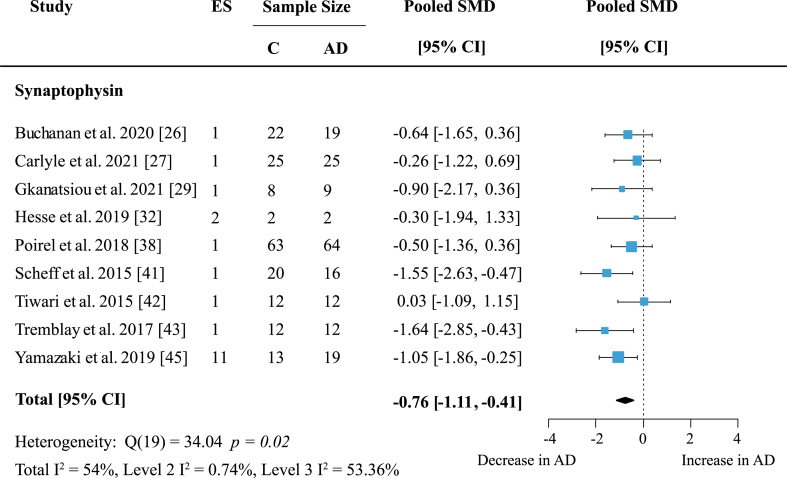
Synaptophysin loss in AD. Forest plot of three-level random effects meta-analysis on synaptophysin levels. A significant decrease in AD (*p* < 0.001) was confirmed. Effect sizes were aggregated to one value per study. Size of effect size symbol represents its weight. Sample sizes represent the maximum number of subjects per group contributing to the analysis for each study. Dashed line depicts no difference between control and AD cohort. AD, Alzheimer’s disease, CI, confidence interval; C, Control; ES, effect size; SMD, standardized mean difference.

The most frequently analyzed presynaptic protein was synaptophysin (SYP) with data available from nine studies. Synaptophysin was measured in seven cortical and five subcortical regions. Meta-analysis confirmed an overall decrease of SYP (Fig. 7: effect size: –0.76; 95% CI: –1.11, –0.41; *p* < 0.001). Overall heterogeneity was moderate due to between-study heterogeneity (Total I^2^: 54%, Level 2 I^2^: 0.74%, Level 3 I^2^: 53.36%) and only one report showed no decrease of SYP levels in AD patients. For exploratory analysis, protein families with low study numbers were also analyzed but none showed significant overall effects (Table 1).

### Function-specific changes in presynaptic proteins

**Fig. 8:**
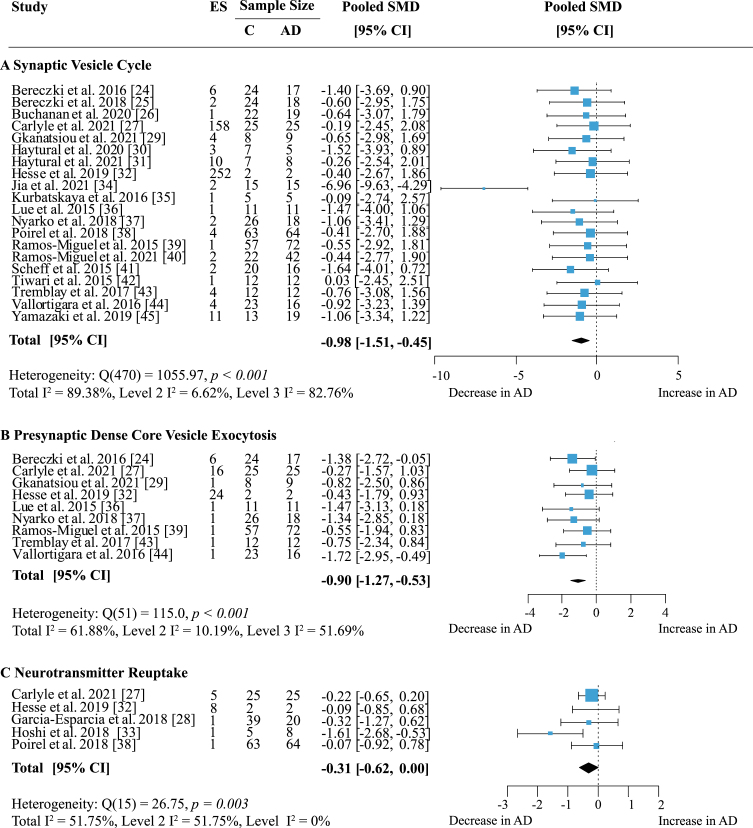
Effect of AD on presynaptic functions. Forest plot of three-level random effects meta-analysis on proteins in different categories of presynaptic functions. There was a significant decrease in proteins involved in the synaptic vesicle cycle (A) (*p* < 0.001), dense core vesicle exocytosis (B) (*p* < 0.001) and neurotransmitter reuptake (C) (*p* = 0.05). Effect sizes were aggregated to one value per study. Size of effect size symbol represents its weight. Sample sizes represent the maximum n per group contributing to the analysis for each study. Dashed line depicts no difference between control and AD cohort. AD, Alzheimer’s disease, CI, confidence interval; C, Control; ES, effect size; SMD, standardized mean difference.

Proteins were grouped according to their presynaptic function and separate meta-analyses were performed. When analyzing specific presynaptic functions, proteins involved in the synaptic vesicle cycle showed the strongest decrease in AD (Fig. 8A; effect size: –0.98; 95% CI: –1.51, –0.45; *p* < 0.001). This functional group also contained the largest number of measurements with 168 proteins in 12 areas from 20 publications. Heterogeneity was high and stemmed mainly from inter-study variations (Total I^2^: 89.38%, Level 2 I^2^: 6.62%, Level 3 I^2^: 82.76%). While most studies reported decreases in protein levels in AD, few showed barely any difference to controls. Only two other functional groups showed significantly reduced levels in AD: dense core vesicle (DCV) exocytosis (Fig. 8B; effect size: –0.90; 95% CI: –1.27, –0.53; *p* < 0.001) and neurotransmitter reuptake (Fig. 8C; effect size: –0.31 95% CI: –0.62, 0; *p* = 0.05). DCV exocytosis had low within-study and moderate between-study heterogeneity (Total I^2^: 61.88%, Level 2 I^2^: 10.19%, Level 3 I^2^: 51.69%) and all reports indicated a moderate to large decrease of protein levels in AD. For neurotransmitter reuptake, heterogeneity was moderate due to intra-study variation (Total I^2^: 51.75%, Level 2 I^2^: 51.75%, Level 3 I^2^: 0%). While a small to moderate decrease of protein expression in AD was most frequently reported, only one study [33] indicated a large loss of proteins compared to controls. Sixteen proteins involved in DCV exocytosis were measured in five brain regions and reported in nine publications; data for neurotransmitter reuptake came from five publications measuring six proteins in four brain areas.

**Table 1:** Summary of secondary analyses

		Number of Studies (k) and sample size				Meta-analysis		Heterogeneity		
	k	n C	n AD	Overall SMD	*p*	Total I^2^	I^2^ Level 2	I^2^ Level 3	*p*_Q_
**Cortical Regions**									
Overall	22	442	433	–1.01[–1.55, –0.47]	< *0.001*	90.57	5.53	85.03	< *0.001*
Frontal Cortex	9	291	286	–0.75[–1.05, –0.45]	< *0.001*	86	80.93	5.07	< *0.001*
Temporal Cortex	6	57	64	–1.04[–1.19, –0.88]	< *0.001*	0	0	0	1
Parietal Cortex^ *^	4	73	72	–0.92[–1.65, –0.19]	*0.014*	86.84	15.18	71.66	< *0.001*
Occipital Cortex^ *^	3	23	30	–0.19[–0.77, 0.39]	0.52	16.42	0	16.42	0.07
HPF^ *^	3	26	25	–0.59[–1.45, 0.27]	0.18	88.44	25	63.44	< *0.001*
Cingulate Gyrus^ *^	3	56	50	–1.07[–1.67, –0.47]	< *0.001*	58.9	0	58.9	0.09
Entorhinal Cortex^ *^	2	27	34	–4.23[–9.17, 0.71]	0.093	95.24	0	95.24	< *0.001*
**Protein Families**									
SNAPs	7	108	93	–0.90[–1.40, –0.40]	< *0.001*	64.87	13.55	51.32	< *0.001*
STXs	6	91	105	–0.20[–0.52, –0.12]	0.21	60.78	60.78	0	< *0.001*
SYTs	5	66	62	–0.27[–0.55, 0.02]	0.07	40.15	40.15	0	*0.01*
SYNs^ *^	4	52	48	–0.58[–1.38, 0.23]	0.16	67.96	0	67.96	*0.04*
SYNGRs^ *^	4	41	40	–0.36[–0.76, 0.04]	0.07	50.75	50.75	0	0.07
VAMPs^ *^	4	53	50	–0.7[–1.7, 0.3]	0.17	85.48	4.89	80.59	*0.001*
**Specific Proteins**									
SYP	9	177	178	–0.76[–1.11, –0.41]	< *0.001*	54.0	0.74	53.36	*0.02*
SNAP25	7	108	93	–1.06[–1.49, –0.63]	< *0.001*	52.46	25.01	27.45	*0.03*
STX1	5	79	93	–0.24[–0.75, 0.27]	0.35	76.55	59.09	17.46	< *0.001*
**Presynaptic Function**									
DCV Exocytosis	9	188	182	–0.9[–1.27, –0.53]	< *0.001*	61.88	10.19	51.69	< *0.001*
NT reuptake	5	134	119	–0.31[–0.62, 0]	*0.05*	51.75	51.75	0	*0.03*
**Regulation of cytosolic calcium levels** ^ *^	4	47	47	–2.04[–5.43, 1.35]	0.24	98.18	0.5	97.68	< *0.001*
**Regulation of presynaptic membrane potential** ^ *^	4	66	60	–1.97[–4.89, 0.94]	0.18	98.01	0.81	97.20	< *0.001*
Presynaptic Signaling Pathway^ *^	2	27	27	–0.4[–0.92, 0.12]	0.13	0	0	0	0.66
Chaperone-mediated protein folding^ *^	2	27	27	–0.09[–0.39, 0.22]	0.58	0	0	0	0.44
NT uptake^ *^	2	27	27	0.36[–0.19, 0.91]	0.2	27.72	27.72	0	0.16
Endocytosis^ *^	2	27	27	–0.4[–0.92, 0.11]	0.13	42.91	39.79	3.12	0.19
SV Cycle	20	398	405	–0.98[–1.51, –0.45]	< *0.001*	89.38	6.62	82.76	< *0.001*
**SV Cycle**									
Exocytosis	19	393	400	–0.86[–1.16, –0.56]	< *0.001*	72.88	22.37	50.51	< *0.001*
Regulation	6	66	61	–0.44[–0.82, –0.05]	*0.03*	60.11	41.36	18.75	< *0.001*
NT loading	6	143	136	–1.3[–3.09, 0.49]	0.16	97.92	4.28	93.64	< *0.001*
Endocytosis^ *^	4	54	51	–0.35[–0.77, 0.06]	*0.09*	46.71	15.73	30.98	< *0.001*
Endosomal Processing^ *^	2	27	27	–0.11[–0.28, 0.07]	0.23	0	0	0	0.69
Clustering^ *^	2	27	27	–0.40[–0.88, 0.08]	0.1	53.61	37.01	16.6	*0.04*
Proton Loading^ *^	2	27	27	–0.3[–0.44, –0.16]	< *0.001*	0	0	0	0.73
Zinc Ion Import^ *^	2	27	27	–0.99[–1.39, –0.60]	< *0.001*	0	0	0	0.29

Significance of meta-analysis result is indicated by *p*, significance of Q-test for heterogeneity is indicated by *p*_Q_. k, Number of studies contributing to analysis; n C, total sample size for control group; n AD, total sample size of AD group; CI, confidence interval; DCV, dense core vesicle; HPF, hippocampal formation; NT, neurotransmitter; SLC, solute carrier family; SMD, standardized mean difference; SNAP, synaptosome associated protein; STX, syntaxin; SV, synaptic vesicle; SYP, synaptophysin; SYN, synapsin; SYNGR, synaptogyrin; SYT, synaptotagmin; VAMP, vesicle-associated membrane protein. ^ *^Fewer than five independent studies available for analysis.

The largest functional group were proteins involved in the synaptic vesicle cycle (SV); individual functions within this group of proteins were also analyzed. Here, proteins involved in synaptic vesicle exocytosis were highly represented with data from 62 proteins in 14 brain areas across 19 studies. Meta-analysis yielded an overall loss in AD subjects (Table 1; effect size: –0.86; 95% CI: –1.16, –0.56, *p* < 0.001) and a moderate heterogeneity due to low within-study and moderate between-study heterogeneity (Total I^2^: 72.88%, Level 2 I^2^: 22.37%, Level 3 I^2^: 50.51%). Twenty-one proteins regulating synaptic vesicle cycles were quantified in six brain regions across six publications. There was a small but reliable overall loss of proteins in this group in AD (Table 1; effect size: –0.44; 95% CI: –0.82, –0.05; *p* = 0.03). Heterogeneity was greatest within studies, but overall moderate (Total I^2^: 60.11%, Level 2 I^2^: 41.36%, Level 3 I^2^: 18.75%). No other functional subgroup showed significant overall effects (Table 1).

Exploratory analysis was also conducted where study numbers were low, and results are presented in Table 1.

## DISCUSSION

Twenty-two publications on presynaptic protein changes in AD patients compared to healthy controls were scrutinized here. Our meta-analysis showed an overall loss of presynaptic proteins in AD. The most prominent protein loss occurred in frontal and temporal cortices. Individual proteins that were highly affected were SNAP25 and synaptophysin, both showing a strong decrease in AD. On a functional level, the most significant decline was observed in proteins involved in exocytosis of dense core vesicles and synaptic vesicles. No evidence for publication bias was observed; all publications were of moderate to high quality. This meta-analysis therefore provides a critical update on the evidence for disruptions of the presynaptic machineryin AD.

Two previous publications have reviewed changes in presynaptic proteins in AD compared to healthy controls. This includes a review by Honer and colleagues [60] and a meta-analysis of synapse counts and synaptic proteins by de Wilde et al. [21]. This latter analysis included 83 studies quantifying synaptic proteins of which a large proportion reported levels of at least one presynaptic marker, resulting in a much higher number of studies contributing to analysis.

The primary analysis included all available protein measurements in all brain regions and revealed a significant loss of presynaptic proteins in AD. Very high between-study heterogeneity was largely due to one study and omission of the data from Jia et al. [34] did not affect the overall study outcome but highlighted the low heterogeneity between and within the studies included in this meta-analysis. The overall lowering of presynaptically expressed proteins in cortical structures therefore confirms the preceding meta-analysis of de Wilde and colleagues and further highlights that this decline expresses protein and brain region specificity [21, 60]. As for the regional specificity, it appears to be an anomaly that neither entorhinal cortex nor hippocampus were confirmed as expressing lower levels of presynaptic proteins despite a wide range of studies that provide compelling evidence for protein pathology during the early onset which increases in severity during late-stage AD [61, 62]. This lack of effect is likely due to the low number of studies and their high heterogeneity, since previous reviews reported some of the strongest effects for these structures based on much higher numbers of reports [21, 60]. Alternative reasons may concern the method of protein quantification, age, and severity of study cohorts and postmortem intervals. An alternative approach would be the inclusion of older publications predating 2015 in order to provide a more complete analysis of the evidence available to date. This would have been against the set limits of this approach, and a more complete analysis for all proteins would then have to be pursued. Suffice to say that the lack of significant synaptic protein loss in hippocampus in this study is due to the small study number and considered to be an anomaly of our analysis. In general, some of the discrepancies between these older meta-analyses/reviews and this work are due to the much richer pool of literature included in their analyses. Nevertheless, the global effects in terms of region and protein specificity were very similar and therefore seem to be robust and reproducible.

Following on from a primary analysis, several separate meta-analyses were performed to explore protein specific changes as well as to determine which presynaptic functions are most affected. SNAP25 was the most affected protein and showed a consistent decline across studies. Apart from some individual proteins engaged in presynaptic structural and transmitter release functions with lower levels in AD patients, the SNAP protein family was the only family of proteins affected significantly by the disease. This is intriguing given the fact that other members of the SNARE complex, including VAMP and syntaxin, strongly involved in the docking of vesicles and rupture of the vesicular membrane enabling transmitter release were not reduced. In some cases (e.g., VAMP) this may be related to the high heterogeneity between studies warranting further examination, but for others the mechanism remains to be explained. When categorized according to presynaptic function, however, proteins involved in cycling of synaptic vesicles also presented with an overall lowering of levels. While this supports our contention that SNARE proteins might more globally be affected by AD pathology, not all elements of vesicle cycling seem equally sensitive to the disease. Most strongly reduced were proteins complexing for exocytosis such as SNARE proteins, complexins or synaptotagmin but also those engaged in vesicle regulation like synapsins and synaptogyrins. However, more studies are needed to confirm these data.

It is difficult to determine to what extent the observed decrease in presynaptic protein levels simply represents global neuronal loss and/or genuine synapse loss. Scheff and collegues [41] used electron microscopy to quantify synapse numbers and, while a global loss of synapses was confirmed in posterior cingulate cortex, there was additional protein loss at surviving synapses. Although only based on two presynaptic proteins, synaptophysin and synapsin-1, the difference in AD compared to controls was higher than for synapse numbers. Also, there was no significant synapse loss in patients with mild cognitive impairment compared to controls, whereas protein loss was similarly significant to the AD cohort. When combining synaptic protein measurements with additional technical approaches(measurement of glutamate transporter VGLUT1, selective axonal labelling), Poirel et al. [38] and Haytural et al. [31] found that both were not affected equally. This would suggest there might be protein loss not simply due to synapse loss as both processes should be affected in similar ratios otherwise. While this may suggest that the presynaptic protein loss could be independent from gross synapse loss this is difficult to ascertain, especially since no publications were available directly comparing synapse numbers to protein quantity.

### Limitations

The current meta-analysis includes a number of potential confounders. 1) Uncertainty on how much effect sizes within one study correlated with each other. Towards this end, sensitivity analysis with different values for effect size correlation was performed, and the direction and magnitude of the overall effect remained very similar and was significant for the whole range of values. We therefore take this as evidence that variable effects size correlations are not a critical limit for the viability of our data and does not play a major role in our subgroup analyses. 2) As multiple publications obtained samples from the same brain banks there may be additional effect size correlations due to the same subjects being analyzed in multiple studies. Here, it was not possible to confirm these as no sample codes were given in the respective publications leading to a lack of information on sample overlap. 3) It is not known how far the use of different methods for protein quantification has influenced the analysis. Even among studies using the same analyses method (for example immunoblotting) differences in loading control markers were frequently observed. Moreover, markers for housekeeping proteins varied between methods which may have affected outcomes. Likewise, the effect of age and differences in postmortem interval between groups within each study and between studies may also affect the findings. These factors could not be accounted for here since epidemiological detail (comorbidities, cause of death) and treatment status (symptomatic AD medications, others) was incompletely reported for each study. However, most publications reported that AD and control subjects were free of overt non-AD related neuropathology or psychiatric disorders. 4) Caution needs to be exercised in generalizing these data to all areas of the brain. Extrapolation from cortex (almost exclusively studied here) to subcortical regions may be particularly problematic, although Yamazaki et al. [45] also found a decrease in synaptic proteins (both pre-and postsynaptic) in most subcortical regions of AD patients that were scrutinized. 5) Protein-specific effects should also be interpreted with caution. as sufficient data from independent studies was available for only a very small subset of proteins which makes it likely that effects of less frequently studied proteins are missed. These issues need to be taken into account when interpreting the findings of the analyses in this study.

### Conclusions

Collectively, our data confirm and extend previous meta-analyses/reviews on the level and distribution of synaptic proteins in postmortem tissue from confirmed advanced stage AD patients. The majority of cortex presents with a lowering of pre-synaptic proteins prior to and independent of frank synapse loss. More fine-grained analyses of the affected transmitter systems and the cortical layers affected is still required. Not all presynaptic proteins are altered equally. SNARE complex proteins and vesicle cycling and recycling peptides are among those most severely reduced, and these are the most likely to be functionally compromised in AD.

## Supplementary Material

Supplementary Material

## Data Availability

All data are available within the paper and its supplementary material.
