# Supplementary material for: A Meta-Analysis on Presynaptic Changes in Alzheimer’s Disease
Source: J Alzheimers Dis. 2024 Jan 2;97(1):145–62. doi: 10.3233/JAD-231034 (PMC10789360; doi:10.3233/JAD-231034)
Supplement: Supplementary Material [file jad-97-jad231034-s001.pdf]

Supplementary Material

A Meta-Analysis on Presynaptic Changes in Alzheimer's Disease

Supplementary Table 1. Data extraction methods.

Study	Available Groups	Included Groups	Sample Size	Age	PMI	% male	Protein data	Additional Notes	ES
**Bereczki et al., 2016 [1][a]**	C, PDD, DLB, AD	C, AD	+	+	+	+	Paper (mean±SD)	All proteins measured with ELISA and WB. Inclusion of ELISA data due to availability of full numerical data	6
**Bereczki et al., 2018 [2]**	C, PDD, DLB, AD	C, AD	+	+	+	+	Paper (mean±SD)	ELISA/ WB validation data[c]	4
**Buchanan et al., 2020 [3]**	C (Braak 0-3), AD (Braak 4-6)	C, AD	+	+	+	+	WBD (scatter plot, mean with 95% confidence interval)	Control group with and without inclusion of subjects with overt non-AD pathology). Control group without comorbid cases was used.	1
**Carlyle et al., 2021 [4]**	Normal, Dementia-AD, Resilient, Frail	Normal, Dementia-AD	+	+	+	+	Paper (raw data)	208 proteins matching presynaptic protein inclusion list were included	208
**Garcia-Esparcia et al., 2018 [5][b]**	Middle-aged controls (MA), AD, DLB	MA, AD	+	+	(+)	+	WBD (bar, mean±SD)		1
**Gkanatsiou et al., 2021 [6]**	C, Pathological Aging, AD, familial AD	C, AD	+	+	+	+	WBD (scatter plot, median and interquartile range)		4
**Haytural et al., 2020 [7]**	C, AD	C, AD	+	+	+	+	WBD (scatter plot, mean)	IHC validation data [e]	3
**Haytural et al., 2021 [8]**	C, AD	C, AD	+	+	+	+	Paper (mean±SD), WBD (scatter plot, mean)	CA and DG layers. Aggregated data was used (pooled ES per protein in CA and DG)	10

**Hesse et al., 2019 [9]**	C (*APOE* ε3/ε4, *APOE* ε4/ε4), AD (*APOE* ε3/ε4, *APOE* ε4/ε4),	*APOE* ε3/ε4 and *APOE* ε4/ε4 pooled for C and AD	+	+	+	+	Paper (raw data)	162 and 161 proteins matching inclusion list in temporal and occipital cortex respectively were included.	323
**Hoshi et al., 2018 [10]**	C, AD	C, AD	+	+	+	+	Paper (mean±SD)	Superior, middle, inferior temporal lobe. Aggregated data (pooled to one ES for temporal lobe)	1
**Jia et al., 2021 [11]**	C, AD	C, AD	+	+	-	+	WBD (bar, mean±SD)	IHC validation data [e]	4
**Kurbatskaya et al., 2016 [12]**	C, Braak II, III, IV, V, VI	C, Braak VI	+	+	+	+	WBD (bar, mean±SEM)	NSE or beta-actin used as loading control. Only NSE data was included	2
**Lue et al., 2015 [13]**	C, Possible AD, AD	C, AD	+	+	-	+	WBD (scatter plot, mean±SEM)		1
**Nyarko et al., 2018 [14]**	C, Early-onset, late-onset AD	C, late-onset AD	+	+	+	+	Author (raw data)	SLC18A2 in different glycosylation states. Total SLC18A2 provided by author was used.	2
**Poirel et al., 2018 [15]**	C, Possible, Probable and Definite AD	C, Definite AD	+	+	+	-	Paper (mean±SEM)		5
**Ramos-Miguel et al., 2015 [16][c]**	Subjects grouped as NCI, MCI, DEM, NIA/Reagan stages or Braak Stages	Braak 0-II as C, Braak V-VI as AD	(+)	(+)	(+)	(+)	WBD (scatter plot, mean±SEM)	STXBP1 short and long splice variant pooled to one overall ES.	1
**Ramos-Miguel et al., 2021 [17][c]**	Subjects grouped as NCI/ MCI/ DEM, dichotomized NIA/Reagan scale	No AD, AD	(+)	(+)	(+)	(+)	WBD (scatter plot, mean±SEM)	WB validation data [e]	2
**Scheff et al., 2015 [18]**	NCI, MCI, AD	NCI, AD	+	+	+	+	WBD (scatter plot, median)		2
**Tiwari et al., 2015 [19]**	C, mild AD (Braak I-II), severe AD (Braak V-VI)	C, severe AD	+	+	+	+	WBD (bar, mean±SEM)		1
**Tremblay et al., 2017 [20]**	NCI, MCI with amyloid pathology, MCI without	NCI, AD	+	+	+	+	Paper (mean± SD)		4

	amyloid pathology, AD								
**Vallortigara et al., 2016 [21]**	C, PDD, DLB, AD	C, AD	+	+	+	+	WBD (bar, mean±SEM)		4
**Yamazaki et al., 2019 [22][d]**	Normal aging, Pathological aging, AD	Normal aging, AD	(+)	(+)	(+)	(+)	Author (mean ± SD)	Dorsolateral prefrontal cortex, oribitofrontal cortex pooled to one ES for frontal cortex	11

Of all data reported in the selected publications, they were predominantly shown in graphs. Numerical values were rarely presented. The Table therefore lists the means by which we extracted the 'Protein data' from each publication under consideration including further demographic information for AD and control groups (Age, PMI, % male subjects, selected cohorts for this meta-analysis from the available study cohorts) and notes on the protein extraction and analysis methods implemented by each author. Also shown are the number of effect sizes the contributed to this meta-analysis. + fully available for each group, (+) partly available, - not available. AD, Alzheimer's disease; CA, *cornu ammonis*; DEM, dementia; DG, dentate gyrus; DLB, dementia with Lewy bodies; ELISA: enzyme-linked immunosorbent assay; ERC, Entorhinal cortex; ES, effect size; IHC, immunohistochemistry; MCI, mild cognitive impairment; NCI, no cognitive impairment; PDD, Parkinson's disease with dementia; PMI, *post-mortem* interval; SD, Standard deviation; SEM, Standard error of the mean; SLC: solute carrier family; STG, Superior temporal gyrus; STXBP: syntaxin-binding protein; WB, western blot; WBD, WebPlotDigitizer.

[a] Data was not available from all subjects for each protein/ area. Demographic characteristics for each group overall.

[b] PMI reported as range.

[c] Demographic Data only available for whole sample.

[d] Demographic data reported as median and range.

[e] Whole-tissue proteomic/ transcriptomic studies with secondary validation method meeting inclusion criteria.

Supplementary Table 2. Study Characteristics.

Study	Brain Bank	Protein	Area	Method	Controls				AD			
					n	Age	% Men	PMI (h)	n	Age	% Men	PMI (h)
**Bereczki et al., 2016 [1]**	BDRN	RAB3A<br>SNAP25	Frontal Cortex, Anterior Cingulate Cortex, Parietal Cortex	ELISA	23-24	79.8 (7.5)	60%	39.1 (23)	14-17	88.1 (7.2)	33%	35 (22.5)
**Bereczki et al., 2018 [2]**	BDRN	SV2C<br>GRIA3<br>GRIA4<br>SYT2	Frontal Cortex<br><br><br>Frontal Cortex	ELISA<br><br><br>WB (GAPDH)	24	80.2 (7.5)	58%	38.8 (23.4)	18	88.1 (7.3)	33%	35 (22.8)
**Buchanan et al., 2020 [3]**	BDRN	SYP	Temporal Cortex	WB (Coomassie)	22	85.3 (6.7)	50%	44.8 (27.8)	19	83.4 (5.1)	58%	46.6 (21.4)
**Vallotigara et al., 2016 [21]**	BDRN	STX1<br>STXBP1<br>VAMP2<br>SNCA	Frontal Cortex	WB (none)	19-23	80.4 (6.9)	58%	37.1 (31.4)	15-16	88.0 (8.0)	31%	34.9 (24.0)
**Kurbatskaya et al., 2016 [12]**	MRC London	SYN1<br>CDK5	Temporal Cortex	WB (NSE)	5	69.2 (19.5)	60%	34.4 (16.2)	5	83 (8.7)	20%	29 (17.7)
**Gkanatsiou et al., 2021 [6]**	Queen Square, London	SNAP25<br>SYP<br>SYT1<br>SYT7	Occipital Cortex	IP-MS	8	75.9 (16.3)	44%	64.1 (22.7)	9	73.4 (9.1)	62%	71.6 (21.6)
**Hesse et al., 2019 [9] [a]**	Edinburgh	162 presynaptic proteins	Temporal Cortex, Occipital Cortex	LC-MS/MS	2 (15)	65.3 (10.4)	80%	69.4 (22.5)	2 (18)	78.6 (9.1)	56%	74.5 (18.6)
**Tiwari et al., 2015 [19]**	King's College London	SYP	Hippocampus	WB (NSE)	12	76.5 (9.9)	50%	21.4 (8.1)	12	75.2 (7.0)	33%	15.1 (6.5)
**Haytural et al., 2021 [8]**	Netherlands	STX1A<br>SYT1<br>SYNGR1<br>VAMP2<br>CPLX1	DG, CA	IHC	7	80.4 (4.3)	0%	6.1 (0.9)	8	80.3 (9.1)	0%	5.1 (1.0)
**Garcia-Esparcia et al., 2018 [5]**	HUB-ICO-IDIBELL, IDIBAPS	SLC1A2	Frontal Cortex	WB (β-actin)	39	61.8 (14.0)	56%	3-9.6	20	80.5 (6.9)	45%	2.5-17.5

**Tremblay et al., 2017 [20]**	ROS	SYP	Parietal Cortex	WB (β-actin)	12	85 (6)	8%	7.4 (6.4)	12	86.1 (5.8)	25%	6.3 (3.9)
**Carlyle et al., 2021 [4]**	ROS, MAP	208 presynaptic proteins	Parietal Cortex	LC-MS3	25	90.5 (5.4)	44%	7.0 (3.0)	25	90.6 (5.5)	42%	7.0 (4.4)
**Ramos-Miguel et al., 2015 [16] [b]**	MAP	STXBP1	Frontal Cortex	WB (β-actin)	57	88.8 (6)	37%	7.2 (4.8)	72			
**Ramos-Miguel et al., 2021 [17] [b]**	ROS, MAP	STX1A STX1B	Frontal Cortex	WB (β-actin)	22	89.4 (6.5)	32%	8.4 (6.0)	42			
**Scheff et al., 2015 [18]**	University of Kentucky, ROS	SYN1 SYP	Posterior Cingulate Cortex	WB (β-actin)	20	87.7 (5.2)	30%	3.0 (1.3)	16	83.8 (7.1)	56%	3.5 (1.5)
**Lue et al., 2015 [13]**	Banner Sun Health	SNAP25	Temporal Cortex	WB (β-actin)	11	85.4 (7.0)	64%	N/A	11	82.4 (8.6)	55%	N/A
**Haytural et al., 2020 [7]**	University of South Carolina	CPLX1 CPLX2 SYNGR1	DG	IHC	7	76.3 (14.8)	57%	10.9 (4.3)	5	70.6 (11.7)	20%	8.0 (3.6)
**Poirel et al., 2018 [15]**	MSBB	SLC17A7 SLC17A6 SLC32A1 SLC1A2 SYP	Frontal Cortex	WB (Ponceau)	63	78.1 (11.1)	N/A	10.9 (7.5)	64	86.7 (9.6)	N/A	6.7 (5.5)
**Yamazaki et al., 2019 [22]**	Mayo Clinic	SYP	Amygdala, Cerebellum, Entorhinal Cortex, Hypothalamus, Occipital Cortex, Parietal Cortex, Posterior Cingulate Cortex, Striatum, Temporal Cortex, Thalamus	ELISA	10-13	57-95	30%		19	62-95	26%	2-24
**Nyarko et al., 2018 [14]**	McGill University	SLC18A2 SNAP25	Frontal Cortex	WB (GSK-3α/β)	26	70.7 (12.5)	46%	19.3 (11.0)	18	83.2 (5.8)	44%	21.4 (9.1)
**Hoshi et al., 2018 [10]**	Brain Research Institute Niigata	SLC1A2	Temporal Cortex	IHC	5	78.2 (2.7)	80%	7 (8.4)	8	80.3 (16.9)	13%	3.6 (0.5)
**Jia et al., 2021 [11]**	Chinese National	GRIK2 GABRB2	Entorhinal Cortex	IHC	15	83.1 (10.2)	47%	N/A	15	89.7 (6.4)	47%	N/A

	Brain Bank, Peking Union Medical College	SLC17A6 CACNB4	

Summary of characteristics for 22 studies, including source of brain samples (Brain Bank), proteins (given in abbreviated form only: for full names of relevant proteins, see main text) and areas analysed, method of protein quantification and demographic characteristics (Age, % Men, PMI) of control and AD groups where available. For western blotting studies, loading control is given in brackets. Demographic data is mean (SD) or range. AD, Alzheimer's disease; BDRN, Brains for Dementia Research Network; CA, *cornu ammonis*; DG, dentate gyrus; ELISA, enzyme-linked immunosorbent assay; IHC, immunohistochemistry; IP-MS, immunoprecipitation mass spectrometry; LC-MS3, liquid chromatography mass spectrometry with quantification at MS3 level; LC-MS/MS, liquid chromatography with tandem mass spectrometry; MAP, Rush Memory and Aging Project; MRC: Medical research council; MSBB, Mount Sinai Brain Bank; PMI, postmortem interval; SD, standard deviation; ROS, Religious Order Study; WB, western blot.

[a] Samples were pooled for protein quantification according to group and *APOE* status, resulting in n=2 per group for meta-analysis.

[b] Demographic characteristics only available for whole sample.

Supplementary Table 3. Sensitivity Analysis.

rho	Overall SMD [95%CI]	p	Overall I^2	I^2 within studies	I^2 between studies	p_Q
**0**	-1.01 [-1.55, -0.47]	*<0.001*	90.55	5.44	85.11	*<0.001*
**0.1**	-1.04 [-1.56, -0.52]	*<0.001*	89.94	6.39	83.55	*<0.001*
**0.3**	-1.04 [-1.54, -0.54]	*<0.001*	89.10	8.46	80.64	*<0.001*
**0.6**	-1.03 [-1.5, -0.56]	*<0.001*	88.08	14.53	73.55	*<0.001*
**0.8**	-1.07 [-1.55, -0.58]	*<0.001*	89.43	28.32	61.11	*<0.001*
**0.9**	-1.10 [-1.61, -0.59]	*<0.001*	91.31	40.53	50.78	*<0.001*
**0.99**	-1.17[-1.73, -0.6]	*<0.001*	93.90	53.76	40.14	*<0.001*

	Overall SMD [95%CI]	p	Overall I^2	I^2 within studies	I^2 between studies	p_Q
**Clustering by research team**	-1.02 [-1.64, -0.4]	*<0.001*	91.74	4.99	86.75	*<0.001*
**Outlier removal (Jia et al., 2021 [11])**	-0.72 [-0.93, -0.52]	*<0.001*	58.73	23.97	34.75	*<0.001*

Overall outcome of meta-analysis when altering specific analysis parameters. *Top:* When using different correlation coefficients (rho) for effect size correlation within studies in the three-level meta-analysis model, the overall outcome did not change substantially. *Bottom:* Clustering on the level of research team instead of study also did not change results. Removing one study with a very large negative effect size, however, did change results: the presynaptic protein loss in AD and heterogeneity on all levels was lower. Significances of meta-analysis result is indicated by p; significance of Q-test for heterogeneity is indicated by p_Q. SMD, standardized mean difference; CI, confidence interval.

Supplementary Figure 1. SNAP loss in AD. Forest plot of three-level random effects meta-analysis on proteins in the SNAP family. SNAPs showed a significant lowering in AD (p < 0.001). To allow visualization of large amounts of data included in analysis, effect sizes were aggregated to one value per study to generate the Forest plot. Sample sizes represent the maximum n per group contributing to the analysis for each study. Dashed line depicts no difference between control and AD cohort. SNAP, synaptosome associated protein; AD, Alzheimer's disease, ES, effect size; C, Control; SMD, standardized mean difference; CI, confidence interval

**Study**	**ES**	**Sample Size**		**Pooled SMD [95% CI]**
		**C**	**AD**	
**Synaptosome associated proteins (SNAPs)**				
Bereczki et al. 2016 [24]	3	24	16	-1.60 [-2.77, -0.43]
Carlyle et al. 2021 [27]	3	25	25	-0.06 [-1.19, 1.08]
Gkanatsiou et al. 2021 [29]	1	8	9	-0.82 [-2.35, 0.72]
Hesse et al. 2019 [32]	6	2	2	-0.46 [-1.81, 0.89]
Lue et al. 2015 [36]	1	11	11	-1.47 [-2.98, 0.03]
Nyarko et al. 2018 [37]	1	26	18	-1.34 [-2.68, 0.01]
Tremblay et al. 2017 [43]	1	12	12	-0.75 [-2.19, 0.69]
**Total [95% CI]**				**-0.90 [-1.40, -0.40]**

Heterogeneity: Q(15) = 48.54, $p < 0.001$

Total I^2 = 64.87%, Level 2 I^2 = 13.55%, Level 3 I^2 = 51.32%

Pooled SMD [95% CI]

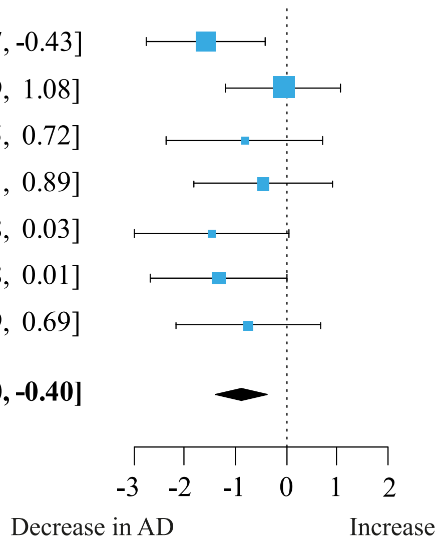

Supplementary Figure 2. Syntaxin proteins unaltered in AD. Forest plot of three-level random effects meta-analysis on proteins in syntaxin family. Syntaxins showed no significant decrease in AD (p = 0.21). To allow visualization of large amount of data included in analysis, ES were aggregated to one value per study to generate the forest plot. Size of effect size symbol represents its weight. Sample sizes represent the maximum n per group contributing to the analysis for each study. Dashed line depicts no difference between control and AD cohort. AD, Alzheimer's disease, ES, effect size; C, Control; SMD, standardized mean difference; CI, confidence interval

Study	ES	Sample Size C	Sample Size AD	Pooled SMD [95% CI]
**Syntaxins**				
Carlyle et al. 2021 [27]	4	25	25	-0.31 [-0.87, 0.24]
Haytural et al. 2021 [31]	2	7	8	0.05 [-0.74, 0.84]
Hesse et al. 2019 [32]	8	2	2	-0.58 [-1.40, 0.23]
Ramos-Miguel et al. 2021 [40]	2	22	42	-0.44 [-1.21, 0.34]
Tremblay et al. 2017 [43]	1	12	12	-0.23 [-1.49, 1.02]
Vallortigara et al. 2016 [44]	1	23	16	1.05 [-0.09, 2.20]
**Total [95% CI]**				**-0.20 [-0.52, 0.12]**

Pooled SMD [95% CI]

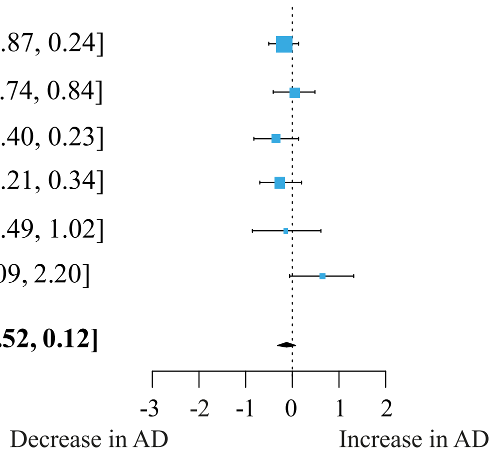

Heterogeneity: Q(17) = 45.02, $p < 0.001$

Total I^2 = 60.78%, Level 2 I^2 = 60.78%, Level 3 I^2 = 0%

REFERENCES

[1] Bereczki E, Francis PT, Howlett D, Pereira JB, Höglund K, Bogstedt A, Cedazo-Minguez A, Baek JH, Hortobágyi T, Attems J, Ballard C, Aarsland D (2016) Synaptic proteins predict cognitive decline in Alzheimer's disease and Lewy body dementia. *Alzheimers Dement* **12**, 1149–1158.

[2] Bereczki E, Branca RM, Francis PT, Pereira JB, Baek JH, Hortobágyi T, Winblad B, Ballard C, Lehtiö J, Aarsland D (2018) Synaptic markers of cognitive decline in neurodegenerative diseases: A proteomic approach. *Brain* **141**, 582–595.

[3] Buchanan H, Mackay M, Palmer K, Tothová K, Katsur M, Platt B, Koss DJ (2020) Synaptic loss, ER stress and neuro-inflammation emerge late in the lateral temporal cortex and associate with progressive tau pathology in Alzheimer's disease. *Mol Neurobiol* **57**, 3258–3272.

[4] Carlyle BC, Kandigian SE, Kreuzer J, Das S, Trombetta BA, Kuo Y, Bennett DA, Schneider JA, Petyuk VA, Kitchen RR, Morris R, Nairn AC, Hyman BT, Haas W, Arnold SE (2021) Synaptic proteins associated with cognitive performance and neuropathology in older humans revealed by multiplexed fractionated proteomics. *Neurobiol Aging* **105**, 99–114.

[5] Garcia-Esparcia P, Diaz-Lucena D, Ainciburu M, Torrejón-Escribano B, Carmona M, Llorens F, Ferrer I (2018) Glutamate transporter GLT1 expression in Alzheimer disease and dementia with Lewy bodies. *Front Aging Neurosci* **10**, 122.

[6] Gkanatsiou E, Nilsson J, Toomey CE, Vrillon A, Kvartsberg H, Portelius E, Zetterberg H, Blennow K, Brinkmalm A, Lashley T, Brinkmalm G (2021) Amyloid pathology and synaptic loss in pathological aging. *J Neurochem* **159**, 258–272.

[7] Haytural H, Mermelekas G, Emre C, Nigam SM, Carroll SL, Winblad B, Bogdanovic N, Barthet G, Granholm A-C, Orre LM, Tjernberg LO, Frykman S (2020) The proteome of the dentate terminal zone of the perforant path indicates presynaptic impairment in Alzheimer disease. *Mol Cell Proteomics* **19**, 128–141.

[8] Haytural H, Jordà-Siquier T, Winblad B, Mulle C, Tjernberg LO, Granholm A-C, Frykman S, Barthet G (2021) Distinctive alteration of presynaptic proteins in the outer molecular layer of the dentate gyrus in Alzheimer's disease. *Brain Commun* **3**, fcab079.

[9] Hesse R, Hurtado ML, Jackson RJ, Eaton SL, Herrmann AG, Colom-Cadena M, Tzioras M, King D, Rose J, Tulloch J, McKenzie C-A, Smith C, Henstridge CM, Lamont D, Wishart TM, Spires-Jones TL (2019) Comparative profiling of the synaptic proteome from Alzheimer's disease patients with focus on the APOE genotype. *Acta Neuropathol Commun* **7**, 214.

[10] Hoshi A, Tsunoda A, Yamamoto T, Tada M, Kakita A, Ugawa Y (2018) Altered expression of glutamate transporter-1 and water channel protein aquaporin-4 in human temporal cortex with Alzheimer's disease. *Neuropathol Appl Neurobiol* **44**, 628–638.

[11] Jia L, Zhu M, Kong C, Pang Y, Zhang H, Qiu Q, Wei C, Tang Y, Wang Q, Li Y, Li T, Li F, Wang Q, Li Y, Wei Y, Jia J (2021) Blood neuro-exosomal synaptic proteins predict Alzheimer's disease at the asymptomatic stage. *Alzheimers Dement* **17**, 49–60.

[12] Kurbatskaya K, Phillips EC, Croft CL, Dentoni G, Hughes MM, Wade MA, Al-Sarraj S, Troakes C, O'Neill MJ, Perez-Nievas BG, Hanger DP, Noble W (2016) Upregulation of calpain activity precedes tau phosphorylation and loss of synaptic proteins in Alzheimer's disease brain. *Acta Neuropathol Commun* **4**, 34.

[13] Lue L, Schmitz CT, Serrano G, Sue LI, Beach TG, Walker DG (2014) TREM2 protein expression changes correlate with Alzheimer's disease neurodegenerative pathologies in post-mortem temporal cortices. *Brain Pathol* **25**, 469–480.

[14] Nyarko JNK, Quartey MO, Heistad RM, Pennington PR, Poon LJ, Knudsen KJ, Allonby O, El Zawily AM, Freywald A, Rauw G, Baker GB, Mousseau DD (2018) Glycosylation states of pre- and post-synaptic markers of 5-HT neurons differ with sex and 5-HTTLPR genotype in cortical autopsy samples. *Front Neurosci* **12**, 545.

[15] Poirel O, Mella S, Videau C, Ramet L, Davoli MA, Herzog E, Katsel P, Mechawar N, Haroutunian V, Epelbaum J, Daumas S, El Mestikawy S (2018) Moderate decline in select synaptic markers in the prefrontal cortex (BA9) of patients with Alzheimer's disease at various cognitive stages. *Sci Rep* **8**, 938.

[16] Ramos-Miguel A, Hercher C, Beasley CL, Barr AM, Bayer TA, Falkai P, Leurgans SE, Schneider JA, Bennett DA, Honer WG (2015) Loss of Munc18-1 long splice variant in GABAergic terminals is associated with cognitive decline and increased risk of dementia in a community sample. *Mol Neurodegener* **10**, 65.

[17] Ramos-Miguel A, Jones AA, Petyuk VA, Barakauskas VE, Barr AM, Leurgans SE, De Jager PL, Casaletto KB, Schneider JA, Bennett DA, Honer WG (2021) Proteomic identification of select protein variants of the SNARE interactome associated with cognitive reserve in a large community sample. *Acta Neuropathol* **141**, 755–770.

[18] Scheff SW, Price DA, Ansari MA, Roberts KN, Schmitt FA, Ikonomovic MD, Mufson EJ (2015) Synaptic change in the posterior cingulate gyrus in the progression of Alzheimer's disease. *J Alzheimers Dis* **43**, 1073–1090.

[19] Tiwari SS, d'Orange M, Troakes C, Shurovi BN, Engmann O, Noble W, Hortobágyi T, Giese KP (2015) Evidence that the presynaptic vesicle protein CSPalpha is a key player in synaptic degeneration and protection in Alzheimer's disease. *Mol Brain* **8**, 6.

[20] Tremblay C, François A, Delay C, Freland L, Vandal M, Bennett DA, Calon F (2017) Association of neuropathological markers in the parietal cortex with antemortem cognitive function in persons with mild cognitive impairment and Alzheimer disease. *J Neuropathol Exp Neurol* **76**, 70–88.

[21] Vallortigara J, Whitfield D, Quelch W, Alghamdi A, Howlett D, Hortobágyi T, Johnson M, Attems J, O'Brien JT, Thomas A, Ballard CG, Aarsland D, Francis PT (2016) Decreased levels of VAMP2 and monomeric alpha-synuclein correlate with duration of dementia. *J Alzheimers Dis* **50**, 101–110.

[22] Yamazaki Y, Shinohara M, Shinohara M, Yamazaki A, Murray ME, Liesinger AM, Heckman MG, Lesser ER, Parisi JE, Petersen RC, Dickson DW, Kanekiyo T, Bu G (2019) Selective loss of cortical endothelial tight junction proteins during Alzheimer's disease progression. *Brain* **142**, 1077–1092.
